# Understanding Walking Behavior among University Students Using Theory of Planned Behavior

**DOI:** 10.3390/ijerph121113794

**Published:** 2015-10-28

**Authors:** Guibo Sun, Ransford A. Acheampong, Hui Lin, Vivian C. Pun

**Affiliations:** 1 Institute of Space and Earth Information Science, The Chinese University of Hong Kong, FYT Bldg., CUHK, Shatin, N.T., Hong Kong, China; E-Mail: gbsun@cuhk.edu.hk; 2 Lab of Interdisciplinary Spatial Analysis, Department of Land Economy, University of Cambridge, 19 Silver Street, Cambridge CB3 9EP, UK; E-Mail: raa49@cam.ac.uk; 3 Department of Geography and Resource Management, The Chinese University of Hong Kong, WFY Bldg., CUHK, Shatin, N.T., Hong Kong, China; 4 Shenzhen Research Institute, The Chinese University of Hong Kong, 2nd Yuexing Road, Nanshan District, Shenzhen 518057, China; 5 Department of Health Sciences, Northeast University, Boston, MA 02115, USA; E-Mail: c.pun@neu.edu

**Keywords:** theory of planned behavior, walking behavior, walking diary, salient belief elicitation, geographic information system, Hong Kong

## Abstract

Walking has been shown to improve physical and mental well-being, yet insufficient walking among university students has been increasingly reported. This study aimed to understand walking behavior of university students using theory of planned behavior (TPB). We recruited 169 undergraduate students by university mass email of the Chinese University of Hong Kong, and first administered a salient belief elicitation survey, which was used to design the TPB questionnaire, to a subset of the study sample. Secondly, all participants completed the TPB questionnaire and walking-oriented diary in a two-day period in December 2012. We mapped the walking behavior data obtained from the diary using geographic information system, and examined the extent to which TPB constructs explained walking intentions and walking behavior using Structural equation model (SEM). We found perceived behavioral control to be the key determinant of walking intention. Shaped by participants’ perceived behavioral control, attitude toward walking and subjective norms, and behavioral intention, in turn had a moderate explanatory effect on their walking behavior. In summary, our findings suggest that walking behavior among university students can be understood within the TPB framework, and could inform walking promotion interventions on the university campuses.

## 1. Introduction

Walking is an important form of physical activity, and it has been shown to improve physical and mental well-being of those who perform it on a regular basis [1]. However, studies have shown that about 40% to 50% of college students are physically inactive [1,2,3]. Although physical inactivity was reported among university students in western countries (e.g., in USA [4,5]), there is a paucity of empirical research on physical activity among university students of East Asian countries [6] (e.g., in Hong Kong [7]). Therefore, understanding the factors influencing walking participation among the general population and university students in particular has become an important public health priority.

The theory of planned behavior (TPB) is a widely used framework that links beliefs and behavior. Its central theme is that intention is the motivational factor that influences behavior; and its constructs (*i.e.*, attitude, subjective norm, and perceived behavioral control) can explain intention and behavior with high accuracy. TPB has been applied to understand the underlying motivations of behavior among different populations [8,9,10], especially students [11,12,13]. For instance, Heath and Gifford demonstrated TPB’s ability to understand bus-riding behavior among Canadian university students [11]; Bamberg *et al.* used TPB to explain the behavioral impacts of offering prepaid bus passes among university students, and concluded that the choice of travel modes (*i.e.*, bus, car, bicycle, and walking) could be affected by interventions that produce changes in the TPB constructs [12,13]. While previous studies suggested TPB having better ability on understanding of physical activity among students than among adults and adolescents [14,15], few studies have used TPB to explicitly model walking behavior among university students.

In this study, we aimed to assess whether TPB explains walking intentions and walking behavior among university students in Hong Kong. While the application of socio-ecological models that capture the complex array of multiple factors influencing health behavior (e.g., walking) have gained prominence in the literature [16,17,18], the application of TPB as the guiding theory is grounded in our quest in the current paper to understand the fundamental psychological consideration underpinning walking behavior among students on campus.

Taking consideration of prior work suggesting that the relevant behavioral outcomes, referents, and control beliefs within the TPB framework are likely to vary among different populations and behavior [19], we conducted a salient belief elicitation survey as the first phase of the study. Understanding how participants perceive a particular behavior through salient belief elicitation could enable better design of a comprehensive TPB-based instrument, and ultimately contribute to tailoring interventions that encourage positive beliefs of that behavior. Despite the importance, the elicitation phase of TPB studies is often neglected by researchers [20,21]. The salient belief elicitation survey in our study contained well-defined open-ended questions, which are critical for establishing the cognitive foundations of a population’s salient behavioral, normative, and control beliefs that are required in TPB [22]. Furthermore, the ability to explain walking behavior may be affected by how behavior is measured. Information absence is also an issue as participants may be unaware of their walking behavior, and unable to describe it accurately [3,15]. Existing literature recognizes the need for a more accurate measurement of walking to improve the veracity of findings of TPB-based walking studies [15,23]. Therefore, in the second phase, we coupled the administration of TPB questionnaires with a walking-oriented diary to provide a more accurate measure of walking behavior to understand such behavior among university students in Hong Kong [24].

## 2. Experimental Section

### 2.1. Study Area, Population, and Research Design

The study was conducted at the Chinese University of Hong Kong (CUHK) in Hong Kong, a hilly yet urban city located on the southern coast of China. The university campus has a complex three-dimension topological layout of pedestrian networks. Express lifts, outdoor escalators, footbridges, and hilly stairs on campus are directly related to different height levels (from 0 to 157 meters). The steeply contoured campus results in the dispersal of buildings and the formation of seven neighborhoods. Each neighborhood houses one of the seven colleges, and is equipped with its own hostels, dining halls, and other ancillary facilities. Recruitment email was sent to all undergraduate students who matriculated at CUHK through CUHK’s Mass Mail System, and 169 students (2.9% of eligible registered university undergraduate students) responded and agreed to participate in the study. The CUHK Survey and Behavioral Research Ethics Committee approved the study. Written informed consent was obtained from all participants.

### 2.2. Salient Belief Elicitation Survey and TPB Questionnaire Design

In order to effectively operationalize the TPB questionnaire, our study began with a salient belief elicitation survey that was administered to 27 volunteers, who were randomly selected from the study sample. The survey contained open-ended and semi-structured interview questions designed to capture participants’ salient beliefs on walking behavior under each TPB construct (Table 1). A think-aloud method was employed to encourage participants to indicate as many factors as they possibly could regarding walking under each TPB construct [25]. The elicitation process continued until saturation was achieved.

A semi-structured TPB questionnaire was developed based on the salient belief elicitation survey. The questionnaire was then pilot tested among the 27 volunteers, and modified after the pilot test to correct any ambiguity wordings. Subsequently, the finalized TPB questionnaire was administered to the study sample on an experimental day in December 2012. The TPB constructs were as the following:

- The attitude construct was formulated as: “For me to use walking as the main transport mode on campus for the next week would overall be: good or bad”. The participants’ attitudinal evaluations derived from the salient belief elicitation survey (*i.e.*, “Environmentally friendly, Comfortable, Pleasant, Less air pollution, Exercise, Have fresh air, Manage stress, Keep fit, Health”) were used to measure attitudes on a 1-to-7 Disagree—Agree response scale. For example, participants were asked “walking as the main travel mode over the next week on campus will be good for my health: disagree—agree”. The subjective probability of behavioral beliefs corresponding to each evaluation item was also measured on a 1-to-7 Unimportant—Important response scale. For example, participants were asked “Health improvement is an important motivation to encourage my walking behavior: unimportant—important”.
- The subjective norm construct was formulated as: (a) The University Authority (e.g. Green Campus Campaign)/(b) my friends in this university/(c) other students within this university “think(s) that I should or should-not choose walking as the main travel mode over the next week on campus” on a 1-to-7 Likely—Unlikely response scale. This was followed by a statement: “With regards to walking, doing what (a) university authority/(b) my friends in this university/(c) other students within this university think(s) I should do is: unimportant—important”.
- To assess perceived behavioral control, participants were asked: (a) “For me to take walk as the main transport mode for the next week on campus would be: difficult–easy” and (b) “My freedom to walk as the main transport mode for the next week on campus is: low—high.”
- Finally, participants also responded to the two intention items: (a) “I intend to walk as the main transport mode for the next week on campus: (b) “I will use walking as the main transport mode for the next week on campus”: unlikely–likely.”

**Table 1:** Open-ended questions for the salient belief elicitation.

*1. Positive or negative feeling about performing the behavior (experimental attitude or affect)* What would you ***like or enjoy*** about walking as the main transport mode over the next week? What would you ***dislike or hate*** about walking as the main transport mode over the next week?
*2. Positive or negative attributes or outcomes of performing the behavior (behavioral beliefs)* What would the ***advantages*** of walking as the main transport mode over the next week be to you? What would the ***disadvantages*** of walking as the main transport mode over the next week be to you? Is there anything **else** associate with your walking as the main transport mode over the next week?
*3. Individuals or groups to whom you might listen who are in favor of or opposed to their performing the behavior (normative referents)* Are there any individuals or groups of people who would ***approve*** of your walking as the main transport mode over the next week? Are there any individuals or groups of people who would ***disapprove*** of your walking as the main transport mode over the next week? Are there ***any other individuals or groups*** who come to mind when you think about walking as the main transport mode over the next week?
*4. Situational or environmental facilitators and barriers that make the behavior easy or difficult to perform (control beliefs and self-efficacy)* What factors or circumstances would make it ***difficult*** for you to walk as the main transport mode over the next week? What factors or circumstances would make it ***easy*** for you to walk as the main transport mode over the next week? Are there ***any other issues*** that come to mind when you think about the difficulty of walking as the main transport mode over the next week?

### 2.3. Measurement of Walking Behavior Using Walking-Oriented Diary

The day following the experimental day, participants were asked to document their walking trips for a 12-hour period from 8:00 am to 8:00 pm on a walking-oriented diary. The diary for this study was adapted from established travel diaries [26], which collected information on the origin and destination of each trip, travel mode choice (walking and vehicular), start and end time, and walked/vehicular routes. Participants were also given a campus map with detailed pedestrian network. Each road segment was assigned a number in the map. Participants were asked to indicate on the map the origin and destination, the specific route(s) taken (recorded using the number sequence of the walked/vehicular road segment), as well as the time spent on walking for each trip. The completed TPB questionnaires and walking diaries were returned to the study headquarter on the third day.

We mapped the walking trips obtained from the walking diary according to road segment numbers using Geographic Information System (ArcGIS 10.0, ESRI, Redlands, CA, USA). Since each participant could have multiple walking trips throughout their 12-hour study period, these repeated measures were classified as “one-to-many” data in ArcGIS. A table join by the “one-to-many” relationship class was conducted in ArcGIS.

### 2.4. Statistical Analysis

Structural equation model (SEM) was used to delineate the chains of mediating causal variables involved in empirical testing of the TPB constructs [12]. The analysis began with two model specifications of the path model, which indicated the relationship between behavior and TPB constructs. We carried out the analysis in a two-step approach [27]. First, the quality of the theoretically derived measurement model was first tested via confirmatory factor analysis (CFA), which established the extent to which the observed indicators provided good and reliable measures (or otherwise) of the latent constructs of the TPB through the use of the squared multiple correlation estimates (SMC). SMC ranges from 0 to 1: the closer the SMC value of an indicator variable to 1, the more reliable it is as a measure of its latent construct [28]. Second, after establishing the reliability of the indicator variables on their respective latent constructs using the identified measurement model, we subsequently proceeded to specify the structural model. The model parameters of the SEM were estimated using maximum likelihood (ML) parameter estimation method [28] since the data were distributed normally with no outliers. In order to derive a composite variable of each belief item within the TPB, we multiplied the score of each belief statement by the corresponding belief evaluation in both of the measurement and structural models [12,13].

The fit between SEM and questionnaire data was evaluated by goodness-of-fit (GFI) adjusted goodness-of-fit (AGFI) and root mean square error of approximation (RMSEA) measures. The GFI and AGFI (ranges from 0 to 1) estimate the degree of fit between the observed data and the theoretical model and AGFI values >0.80 indicate an acceptable fit [28]. The RMSEA index compensates for sample size, with low values (≤0.08) indicating an acceptable fit. AMOS 21 in SPSS was used for all analyses.

## 3. Results and Discussion

### 3.1. Descriptive Analysis

The mean age of the 169 participants was 18.6 years old (standard deviation = 1.3). The sample was evenly distributed by gender (56% female), which was representative of the gender distribution at the CUHK. Three walking behavior measures were created from the walking-oriented diary: walking distance, walking ratio (the number of a participant’s daily walking trips divided by the number of total (walking and vehicular) daily trips), and walked altitude range. No gender differences were present on the walking behavior outcomes. Descriptive statistics of these measures are shown in Table 2.

**Table 2:** Descriptive analysis of walking behavior (*N* = 169).

Measures of Walking Behavior	Mean ± Standard Deviation	Median	Minimum	Maximum
Walking distance (meters per day)	3290 ± 1669	3235	281	9541
Walking ratio	0.90 ± 0.09	0.92	0.56	1
Walked altitude range (meters per day)	99 ± 35	102	11	149

**Figure 1:**
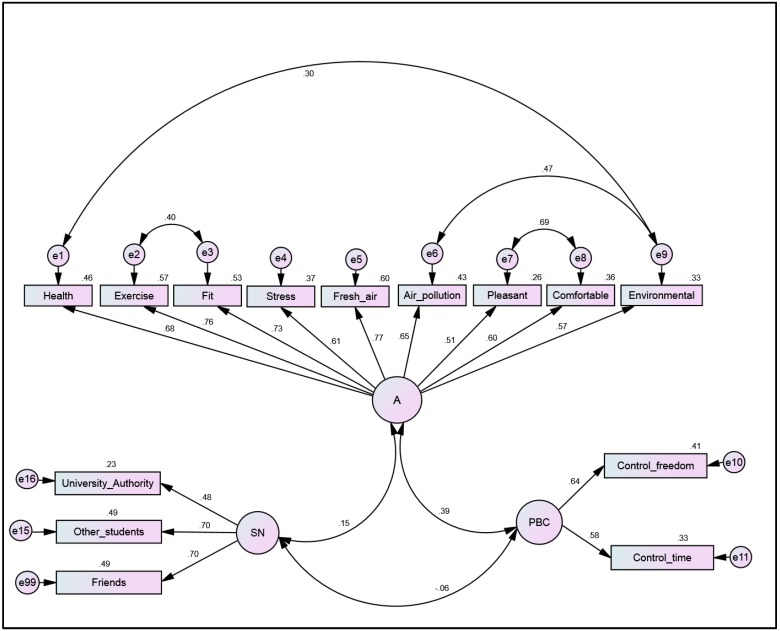
Confirmatory factor analysis of TPB constructs. Notes: Model identification indexes: ${\text{χ}}^{2}$ = 121.50 (df = 70); *p* < 0.01; RMSEA = 0.073; GFI = 0.91; AGFI = 0.83). A = Attitude; PBC = perceived behavioral control; SN = Subjective norm.

### 3.2. Cognitive Mechanisms Underlying Walking Behavior

Three latent factors were obtained in the measurement models (Figure 1), namely (a) attitude measured by nine observed indicators and (b) perceived behavioral control measured by two indicators, and (c) subjective norm measured by three indicators. The specified measurement model in CFA was identified based on the prescribed SEM model identification indexes (${\text{χ}}^{2}$ = 121.50 (degrees of freedom, df = 70); *p* < 0.01; RMSEA = 0.073; GFI = 0.91; AGFI = 0.83). The model showed that all the indicators of attitude provided reliable measures with SMC values ranging between 0.26 and 0.60. The observed measures of participants’ perceived behavioral control provided reliable estimates with SMC values ranging between 0.33 and 0.41. Similarly, in the case of subjective norm construct, participants’ perception of the influence of university authority (SMC = 0.23), other students (SMC = 0.49), and friends (SMC = 0.49) performed reliably as indicator measures. All of the SMC are shown on top of the rectangles containing the indicator variables in Figure 1.

**Figure 2:**
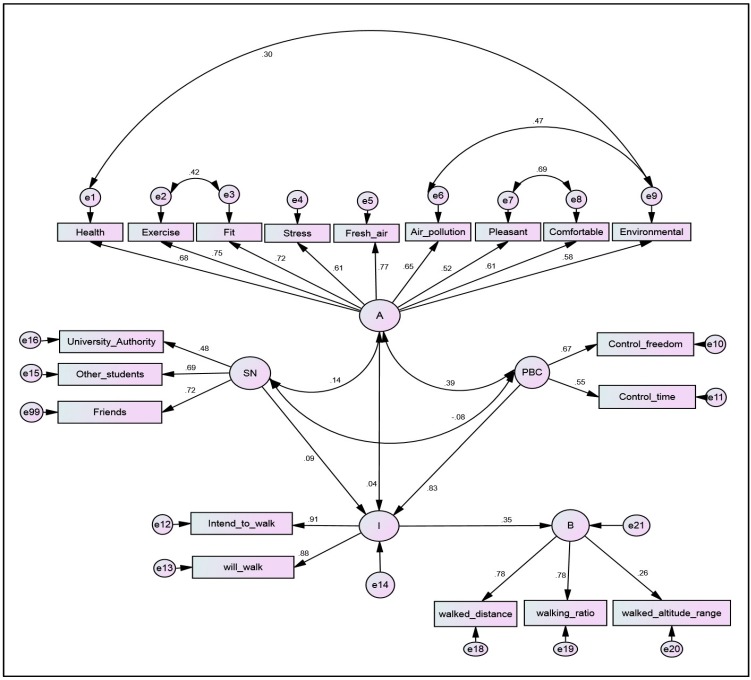
Structural equation model of the TPB survey on walking behavior. Notes: Model identification indexes: ${\text{χ}}^{2}$ = 219.42 (df = 141); *p* < 0.01; RMSEA = 0.064; GFI = 0.91; AGFI = 0.81). A = Attitude; PBC = perceived behavioral control; SN = Subjective norm; I = Intention; B = Behavior.

As shown in Figure 2, the structural model of SEM comprises of all the latent constructs in the CFA in addition to the measures of participant’s walking intentions and walking behavior. The identification of structural model implies that the hypothesized direction of effects among the model variables was supported by the data (Model identification indexes: ${\text{χ}}^{2}$ = 219.42 (df = 141); *p* < 0.01; RMSEA = 0.064; GFI = 0.91; AGFI = 0.81). Each TPB construct (e.g., attitudes, subjective norms and perceived control) was hypothesized to affect walking intentions directly. These TPB constructs, in turn, affected walking behavior through walking intention.

The relative effect sizes of each construct on walking intentions, and of how intentions affect walking behavior are shown in Table 3. Perceived behavioral control had the largest positive and significant influence on walking intentions (standardized path coefficient = 0.826, *p* < 0.01). Subjective norm had a smaller and statistically insignificant positive influence on intentions (standardized path coefficient = 0.091, *p* = 0.394), followed by the influence of attitudes on intentions (standardized path coefficient = 0.043, *p* = 0.723). The correlation and covariance estimates of all latent constructs are shown in Table 4. Participants’ perceived control was moderately correlated with attitudes (correlation = 0.39; *p* = 0.005). The magnitudes of standardized effects (e.g., total, direct, and indirect effects) among the latent constructs in the SEM are shown in Table 5.

**Table 3:** Maximum likelihood parameter estimates for the SEM.

Parameters			b	S.E.	B	C.R.	*p*
Health	<---	A	0.788	0.117	0.682	6.715	<0.01 **
Exercise	<---	A	0.99	0.137	0.751	7.202	<0.01 **
Fit	<---	A	0.904	0.131	0.717	6.927	<0.01 **
Stress	<---	A	0.908	0.148	0.61	6.124	<0.01 **
Fresh air	<---	A	0.972	0.131	0.773	7.393	<0.01 **
Air pollution	<---	A	1		0.654		
Pleasant	<---	A	0.622	0.117	0.521	5.323	<0.01 **
Comfortable	<---	A	0.68	0.111	0.61	6.125	<0.01 **
Environmental	<---	A	0.74	0.094	0.58	7.907	<0.01 **
Control freedom	<---	PBC	1		0.666		
Control time	<---	PBC	0.756	0.151	0.552	5.001	<0.01 **
University authority	<---	SN	0.573	0.143	0.477	4.002	<0.01 **
Other students	<---	SN	1		0.693		
Friends	<---	SN	0.964	0.237	0.716	4.062	<0.01 **
Walked distance	<---	B	17885.803	4233.236	0.784	4.225	<0.01 **
Walked altitude range	<---	B	123.982	48.153	0.259	2.575	0.01 *
Walking ratio	<---	B	1		0.78		
Intent to walk	<---	I	0.959	0.084	0.883	11.394	<0.01 **
Will walk	<---	I	1		0.913		
I	<---	SN	0.016	0.018	0.091	0.852	0.394
I	<---	PBC	0.14	0.034	0.826	4.076	<0.01 **
I	<---	A	0.006	0.018	0.043	0.355	0.723
B	<---	I	0.021	0.006	0.352	3.243	<0.01 **

Notes: A = Attitude; PBC = perceived behavioral control; SN = Subjective norm; I = Intention; B = Behavior; S.E. = Standard Error; C.R. = Critical Value; b = Unstandardized path coefficients; β = Standardized path coefficients; ** *p* < 0.01; * *p* < 0.05.

**Table 4:** Maximum likelihood estimates of covariance and correlations for the SEM.

Parameters			Covariance	S.E.	Correlation	C.R.	*p*
A	PBC	<--->	23.541	8.3	0.39	2.836	0.005 *
A	SN	<--->	8.673	6.832	0.145	1.269	0.204
PBC	SN	<--->	−4.169	7.387	−0.078	−0.564	0.572
e7	e8	<--->	42.025	6.975	0.687	6.025	<0.01 **
e6	e9	<--->	37.911	8.191	0.466	4.628	<0.01 **
e2	e3	<--->	21.806	6.428	0.42	3.392	<0.01 **
e1	e9	<--->	17.731	5.285	0.298	3.355	<0.01 **

Notes: A = Attitude; PBC = perceived behavioral control; SN = Subjective norm; I = Intention; B = Behavior; S.E. = Standard Error; C.R. = Critical Value; e = error terms; ** *p* < 0.01; * *p* < 0.05.

**Table 5:** Standardized total effects, direct, and indirect effects derived from the SEM.

**Total Effects**					
	PBC	A	SN	I	B
I	0.826	0.043	0.091	0	0
B	0.29	0.015	0.032	0.352	0
**Direct Effects**					
	PBC	A	SN	I	B
I	0.826	0.043	0.091	0	0
B	0	0	0	0.352	0
**Indirect Effects**					
	PBC	A	SN	I	B
I	0	0	0	0	0
B	0.29	0.015	0.032	0	0

Notes: A = Attitude; PBC = perceived behavioral control; SN = Subjective norm; I = Intention; B = Behavior.

Overall, walking intentions, shaped by the perceived behavioral control, attitudes, and subjective norms of the participants had a moderate influence on their walking behavior (standardized path coefficient = 0.36, *p* < 0.05). The structural model’s SMC estimate of the direct effect of attitudes, perceived control and subjective norm, on intentions was 0.71. Moreover, the SMC estimates of all the four latent constructs on participants’ walking behavior were moderate (SMC = 0.13). These indicate that the TPB constructs alone yielded a moderate explanatory effect on participants’ walking behavior. In the SEM analysis, we also found that perceived behavioral control had a negative and insignificant influence on walking behavior (standardized path coefficient = −0.002, *p* = 0.55), thus we did not include the direct effect from PBC to walking behavior in the final model.

### 3.3. Discussions

In this study, we found that TPB provides a theoretical foundation for designing structured survey instruments to understand the cognitive mechanisms underlying behavioral intentions and walking behavior observed on a university campus in Hong Kong. This is consistent with previous findings on the understanding walking behavior in adult populations using TPB [15,29,30]. Our findings shows that TPB constructs provided a moderate explanatory effect on the study participants’ walking behavior. Among the latent constructs, only perceived behavior control had a large positive and statistically significant influence on participants walking intentions—a proximal determinant of walking behavior. Overall, the influence of attitudes, subjective norm, perceived behavioral control, and intentions on walking behavior was moderate.

Our finding is consistent with empirical work of [3] that found perceived behavioral control to be the strongest predictor of walking intentions and walking behavior. Moreover, Scott *et al.* [15] similar to our findings, also reported that perceived behavioral control remained the only significant contributor of walking behavior within a small sample of students (*N* = 41). However, our findings differ from those reported by Galea and Bray, who found a moderate effect of perceived behavioral control on walking with only 8% of the attributed variance in walking behavior [30]. Participants in our current study perceived walking as a daily physical activity without significant constraints, whereas participants in Galea and Bray’s study, who suffered debilitating effects of intermittent claudication condition, perceived the contrary.

These findings might have implications for walking promotion interventions targeting students on the university campus. For instance, we found that subjective norm did not exert a statistically significant influence on walking intentions and behavior, which suggests that the judgment of significant others did not influence students’ walking. This is in line with existing empirical works that showed subjective norm notably explaining less variance [8,20] or having a negative contribution to walking [15]. Findings from the current study suggest that the ongoing persuasion-based Green Campus campaign led by the CUHK authority may not be an effective walking promotion intervention. We also reported that the influence of participants’ beliefs on the overall physical, mental health, and environmental benefits (*i.e.*, attitudes) on walking intention were not as strong and statistically significant as their perception of the ease with which walking as a physical activity could be carried out on a daily basis on campus (*i.e.*, perceived control). The findings suggest that walking promotion interventions could be anchored on participants’ stronger perception of walking as an easy and flexible mode of transportation on campus. Interventions could also be focused on creating awareness of the overall personal health and environmental sustainability benefits associated with walking to encourage students to adopt walking as a mode of transport on campus, given that attitude was moderately and positively correlated with perceived behavioral control. A comprehensive approach might include designating pedestrian walkways to ensure the safety of walkers, providing street furniture, and aligning walking paths across areas of scenic beauty that provides a balance between shade and sunlight.

### 3.4. Limitations

Our study has limitations. First, the walking-oriented diary template was adapted from established travel diaries [26], but was assessed for validity by colleagues in Urban Planning and Geography at CUHK, and pilot tested. The TPB questionnaires and walking diary were not standardized. Hence, modifications might be needed if used in other contexts. In addition, the measures of walking behavior in this study were based on a one-day walking diary record. Although recording walking patterns over several days (e.g., one week) could improve observed walking behavior outcomes, in this study, the use of a detailed walking-oriented diary made it difficult and time consuming on the part of the study participants to do so. Second, our findings may not be generalizable to other student populations, since we only included a single university in Hong Kong. In addition, the study focused mainly on the cognitive aspects of walking behavior using TPB variables to understand walking behavior. The interplay between the cognitive and other physical factors determining walking behavior warrants further exploration. Third, we acknowledge that it is equally important to extend the TPB or adopt other theoretical frameworks such as socio-ecological models that would allow capturing the effect of factors including habits, unconscious behavior, group behavior, learned experiences, and mobility socialization through generations on walking behavior. Last but not least, our sample size is small, which did not permit more complex model specifications involving other factors including level of weight, lifestyle, or socioeconomic indicators (e.g., household income) that may influence walking behavior either directly or indirectly as mediators.

Despite these limitations, this study also has several strengths. We showed that TPB could provide a theoretical foundation for designing structured survey instruments, and help explaining the cognitive mechanisms underlying observed walking behavior. Our study has contributed to the rather limited literature on walking behavior from contexts outside of Europe and the Americas. The accretion of knowledge on walking behavior of students from our current study would provide an empirical reference for walking promotion interventions targeting university students in CUHK, and other university campuses alike in Hong Kong and elsewhere.

## 4. Conclusions

The cognitive considerations underlying walking behavior among university students in Hong Kong could be understood using TPB framework. In this study, we found perceived behavioral control to be a key determinant of walking intentions—a proximal determinant of walking behavior. Behavioral intention, shaped by participants’ perceived behavioral control and attitude toward walking, had a moderate influence on walking behavior, while that of attitude and subjective norms was small and statistically insignificant. This study suggests that public health practitioners and urban planners could apply TPB to develop walking promotion interventions for university students.
